# Nonsteroidal Anti-inflammatory Drug Interaction with Prostacyclin Synthase Protects from Miscarriage

**DOI:** 10.1038/s41598-017-10150-2

**Published:** 2017-08-29

**Authors:** Digna R. Velez Edwards, Todd L. Edwards, Michael J. Bray, Eric Torstenson, Sarah Jones, Martha J. Shrubsole, Harvey J. Muff, Katherine E. Hartmann

**Affiliations:** 1 Vanderbilt Epidemiology Center, Vanderbilt University, Nashville, Tennessee 37203 USA; 2 Institute for Medicine and Public Health, Vanderbilt University, Nashville, Tennessee 37203 USA; 3 Department of Obstetrics and Gynecology, Vanderbilt University, Nashville, Tennessee 37203 USA; 4 Vanderbilt Genetics Institute, Vanderbilt University, Nashville, Tennessee 37203 USA; 5 Division of Epidemiology, Vanderbilt University, Nashville, Tennessee 37203 USA; 6 Division of General Internal Medicine and Public Health, Vanderbilt University, Nashville, Tennessee 37203 USA; 7 GRECC, Department of Veterans Affairs, Tennessee Valley Healthcare System, Nashville, 37203 Tennessee USA

## Abstract

This study evaluates the relationship between single nucleotide polymorphisms (SNPs) in nonsteroidal anti-inflammatory drug (NSAID) metabolism and related pathways and spontaneous abortion (SAB, gestation < 20 weeks) risk. Women were enrolled in *Right from the Start *(2004–2010) prospective cohort. Periconceptional NSAIDs reported through the sixth week of pregnancy were obtained from study interviews. We evaluated 201 SNPs in 600 European American women. Interaction analyses between NSAID use and SNPs were conducted using logistic regression, adjusted for confounders. We also evaluated prostaglandin E2 urinary metabolite (PGE-M) in an independent population for association with SNPs using linear regression. NSAID use was reported by 63% of cases and 62% controls. The most significant interaction was at prostacyclin synthase (*PGIS*) rs5602 (OR = 0.34, 95% CI 0.19–0.60, p = 2.45 × 10^−4^) and was significant after a Bonferroni correction. NSAID users were protected from SAB (OR = 0.78, 95% CI 0.56–1.10), while non-NSAID users were at increased risk (OR = 2.11, 95% CI 1.35–3.29) in rs5602 stratified analyses. rs5602 also associated with increased PGE-M levels (Beta = 0.09, 95% CI −0.002–0.19, p = 0.033). We identified an association between a *PGIS* variant and SAB risk that is modified by NSAIDs use during pregnancy and directly associated with increased levels of PGE metabolites. This suggests the potential use of genetic information to guide pharmaceutical intervention to prevent adverse pregnancy outcomes.

## Introduction

Non-steroidal anti-inflammatory drugs (NSAIDs) are one of the most common medication exposures reported during the first trimester of pregnancy^1^. Over 40% of women of reproductive age report NSAID use^1^. NSAID use during preconception and early pregnancy have been associated with an estimated 2.7–7.0 fold increase risk for spontaneous abortion (SAB, pregnancy loss <20 weeks gestation)^2–4^. However, the majority of published studies of pregnancy NSAID use were limited by recall bias, exposure misclassification, limited information about the timing of NSAID exposure, and poor understanding of the timing of effects of NSAID use on risk of SAB. Our group and others have also evaluated the relationship between NSAID exposure and SAB risk finding no population-level effect of NSAIDs on risk^1, 5^. In addition, our group has observed a disparity between African Americans and European Americans regarding the risk of SAB given NSAID use, with African Americans having reduced risk when exposed and European Americans having no change in risk^1, 6^. Additionally gene variants in NSAID pathways have both associated risk for disease and been reported to interact with NSAID use to modify risk for disease in prior studies^7, 8^. This supports a potential genetic basis for the observed differences. No study to date has comprehensively examined how individual genetic variation can lead to differences in patient responses to NSAIDs and how this influences risk for SAB.

**Figure 1:**
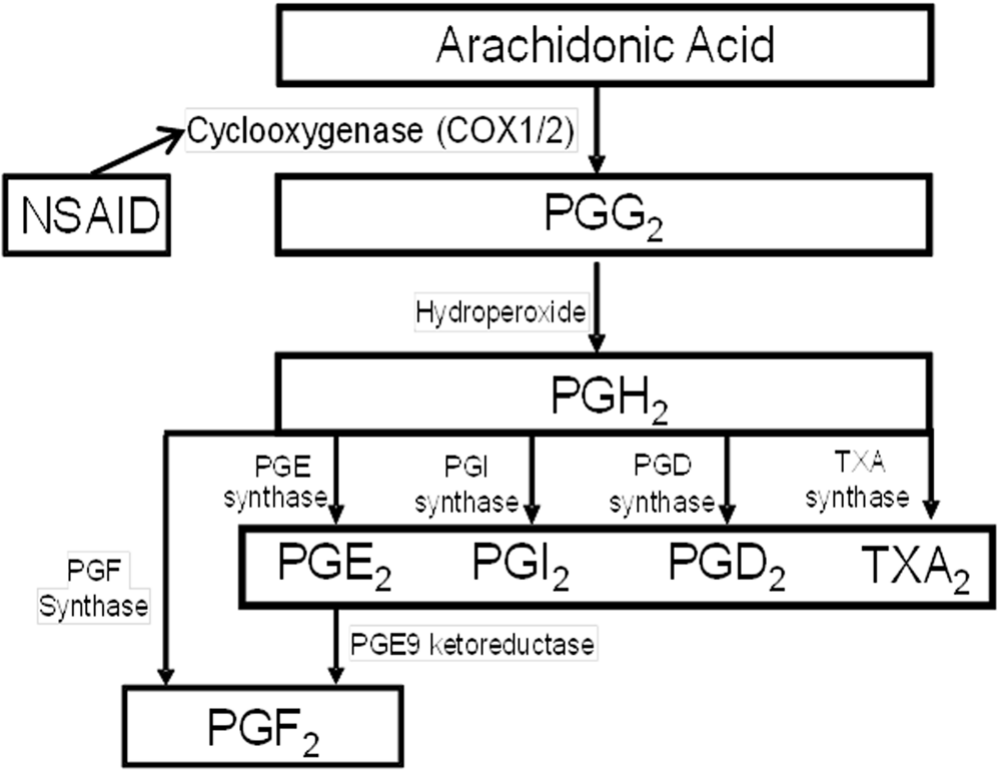
Prostaglandin metabolism and NSAID use. This figure shows the metabolism of arachodonic acid and the role of Cox 1/2 and NSAIDs in the process.

The biological basis for suspecting a link between NSAIDs and risk of SAB rests on the multiple stages in fetal development that involve prostaglandin (PG) synthesis, particularly during early pregnancy when PG enzymes, such as PG E synthases (PGE), may be essential for establishing the implantation and early placentation. In addition, PGE2 and other proinflammatory cytokines have been shown to be overexpressed in endometrial tissue of women who experienced SABs^9–11^. NSAIDs inhibit COX enzymes, which catalyze the formation of PGs from arachidonic acid and thus reduce PG synthesis (Fig. 1). PGs have important roles in both maintaining and achieving pregnancy^12^ and altering levels has direct effects on conception, implantation, and maintenance of pregnancy in animal models^13–23^.

NSAIDs have direct effects on PG pathways and pregnancy, and evidence from epidemiologic and pharmacogenomic studies support a genetic predisposition to individual drug response. This study examines the interactions between NSAID use and single nucleotide polymorphisms (SNPs) from NSAID metabolic and related pathways for association with SAB risk. Due to the known relationship between PGE2 and SAB in prior gene expression studies, we also perform secondary analyses to further evaluate whether most statistically significant SNPs has functional significance by investigating their association with PGE2 urinary metabolites (PGE-M) in an independent population.

## Results

We limited our analyses to subjects of European American ancestry. We included 600 European American RFTS subjects in analyses. RFTS study characteristics are presented on Table 1. The mean age across SAB cases and controls was 31 ± 5 and 30 ± 4, respectively. The majority of subjects had normal body mass index (BMI) (20–25), a household income greater than $40,000, no history of preterm birth or SAB, were married, and non-smokers. SAB cases were also more likely to have parity of one or more than those who did not experience a loss (parity ≥ 1 SAB cases 55%; without loss 49%). The majority of the subjects were enrolled in Tennessee (74%); however, more controls (83%) than cases (48%) were recruited from Tennessee. NSAID use was reported for 63% of SAB cases and 62% of controls. Among NSAID users, the most common type of NSAIDs used were proprionic acid derivatives (89% SAB cases and 81% controls) (Table 2).

**Table 1:** Study characteristics of European American participants by pregnancy outcome.

**Variable**	**N**	**SAB (n = 165)**	**Without Loss (n = 435)**
**NSAID Use (%)**			
Yes	351	63	62
No	217	37	38
**Maternal age (mean yrs ± S.D.)**	600	31 ± 5	30 ± 4
**BMI (mean ± S.D.)**	583	25 ± 5	25 ± 6
**BMI Categorical (%)**			
Underweight (<20)	55	9	9
Normal weight (20 to <25)	296	47	50
Overweight (25 to <30)	138	24	23
Obese (≥30)	94	20	18
**History of spontaneous abortions (%)**			
0	433	73	80
≥1	123	27	20
**History of induced abortions (%)**			
0	507	91	91
≥1	49	9	9
**History of preterm birth (%)**			
0	511	91	92
≥1	45	9	8
**Parity (%)**			
0	274	45	51
≥1	282	55	49
**Household income (%)**			
<$40,000	89	13	17
≥$40,001 to <$80,000	235	40	44
≥$80,000	226	47	39
**Smoking status (%)**			
Never	425	78	76
Current	7	2	1
Former	122	20	23
**Study site (%)**			
North Carolina	159	52	17
Tennessee	441	48	83

BMI-body mass index, kg/m^2^.

**Table 2:** Major NSAID classes by SAB outcome reported at 5% or more in *Right from the Start*, 2004–2010.

NSAID Class	N^†^	SAB %	Without Loss %
		(n = 165)	(n = 435)
Salicylates	35	11	10
Para-aminophenol derivatives	25	7	7
Propionic acid derivatives*	292	89	81

^†^Participants could have reported more than one NSAID and therefore could be included in more than one NSAID class. As a result total percentages across classes do not sum to 100%. Percentages represent total N for NSAID class divided by total number of NSAIDs by status.

In order to determine the relationship between SAB risk, SNPs, and NSAID exposure we first evaluated NSAID by SNP interactions, and then followed those analyses with single SNP association analysis with SAB risk, stratifying by NSAID exposure. The strongest NSAID by SNP interactions were observed for genes within the Cox-1 and -2 signaling pathways. The strongest associated gene was PGI2 synthase (*PGIS*, also known as prostacyclin synthase), with the smallest p value = 2.45 × 10^-4^ (OR = 0.34, 95% CI 0.19–0.60 Table 3). This association was statistically significant after a Bonferroni correction for multiple testing. The SNP rs5602 is located in the 3′ UTR region of *PGIS*. The second strongest association from the NSAID by SNP interaction analyses was *CYP2A6* in NSAID use (rs8192729, OR = 0.20, 95% CI 0.07–0.56, p = 2.33 × 10^−3^, Table 3). Several NSAID by SNP interactions were observed in PGD2 Receptor (*PGDR*) with p-values ranging from 6.15 × 10^−3^ to 0.031 (Table 3). We also observed nominally significant (p < 0.05) single SNP associations at several other SNPs in *PGIS*.

**Table 3:** Strongest SNPxNSAID interaction in RFTS.

Chr	Gene	Location	rsID	BP	MA	MAF		OR_intxn_^1^	95% CI		P
						Cases	Controls		Lower	Upper	
20	*PGIS*	3′-UTR	rs5602	47555385	A	0.48	0.46	0.34	0.19	0.60	2.45 × 10^–4^
20	*PGIS*	Intron	rs576119	47560389	G	0.26	0.30	2.78	1.46	5.30	1.94 × 10^–3^
19	*CYP2A6*	Intron	rs8192729	46042836	A	0.08	0.08	0.20	0.07	0.56	2.33 × 10^−3^
20	*PGIS*	Intron	rs6090996	47567189	A	0.23	0.20	3.11	1.47	6.59	2.99 × 10^−3^
20	*PGIS*	Intron	rs522962	47576769	A	0.45	0.47	0.44	0.25	0.77	3.69 × 10^−3^
14	*PGDR*		rs11623990	51802887	A	0.22	0.21	2.72	1.33	5.58	6.15 × 10^−3^
20	*PGIS*	C- > A Synonymous	rs5629	47563113	A	0.23	0.27	2.46	1.27	4.78	7.78 × 10^−3^
20	*PGIS*	Intron	rs6019876	47563394	G	0.23	0.27	2.40	1.24	4.65	9.47 × 10^−3^
14	*PGDR*	Intron	rs4898758	51806518	G	0.14	0.14	2.80	1.20	6.54	0.018
14	*PGDR*		rs12885771	51814024	A	0.14	0.14	2.70	1.18	6.20	0.019
20	*PGIS*		rs693649	47621486	A	0.18	0.19	2.34	1.10	4.97	0.028
14	*PGDR*	Intron	rs11157908	51806060	A	0.14	0.12	2.58	1.09	6.08	0.031
20	*PGIS*		rs574113	47553590	G	0.36	0.31	0.52	0.28	0.97	0.038

Chr-chromosome; BP-base pair; MA-minor allele; MAF-minor allele frequency; OR-odds ratio; CI-95% Confidence Interval.

^1^Models adjusted for NSAID use, age, BMI, SAB history, and ancestry.

We further performed secondary NSAIDxrs5602 interaction analyses limiting SAB cases to those with losses <10 weeks and ≥10 weeks to assess whether the timing of the NSAID use, with regard to gestational age, had a different impact on SAB risk. We also performed secondary analyses excluding NSAID users who used products that had aspirin (e.g. salicylates) as a secondary ingredient. We did observe a slight strong effect among losses that occurred <10 weeks compared to those that occurred at ≥10 weeks (Table 4), however, the effect sizes for each analysis were included in both set of confidence intervals (<10 weeks NSAIDxrs15602 OR = 0.36, 95% CI 0.18–0.72, p = 3.93 × 10^−3^; ≥10 week NSAIDxrs5602 OR = 0.48, 95% CI 0.22–1.05, p = 0.066). Additionally, analyses excluding salicylates were still statistically significant (Table 4, NSAISxrs5602 interaction OR = 0.41, 95% CI 0.23–0.72, p = 1.95 × 10^−3^).

**Table 4:** *PGIS* rs5602 stratified by case <10 weeks and ≥10 weeks.

rsID	OR_intxn_^1^	95% CI		P
		Lower	Upper	
rs5602 SAB <10 weeks	0.36	0.18	0.72	3.93 × 10^−3^
rs5602 SAB ≥10 weeks	0.48	0.22	1.05	0.066
rs5602 excluding aspirin^2^	0.41	0.23	0.72	1.95 × 10^−3^

OR-odds ratio; CI-95% Confidence Interval.

^1^Models adjusted for NSAID use, age, BMI, SAB history, and ancestry; ^2^Excluded any NSAID that included aspirin, such as salicylates.

We then evaluated the statistical correlations between the SNPs (linkage disequilibrium [LD], reported as r^2^ and D’) we included in analyses within *PGIS*, in order to assess whether they represented a single association signal or multiple signals. The majority of the *PGIS* SNPs (seven SNPs) that interacted with NSAID use were in or nearby one LD block that had moderate to strong D’ in both cases and controls, but weaker r^2^ (Supplemental Fig. 1). The second LD block consisted of *PGIS* rs477627 and rs693649, these SNPs had a D’ = 1 in controls but weaker D’ in cases and overall weak r^2^ (Supplemental Fig. 1).

**Figure 2:**
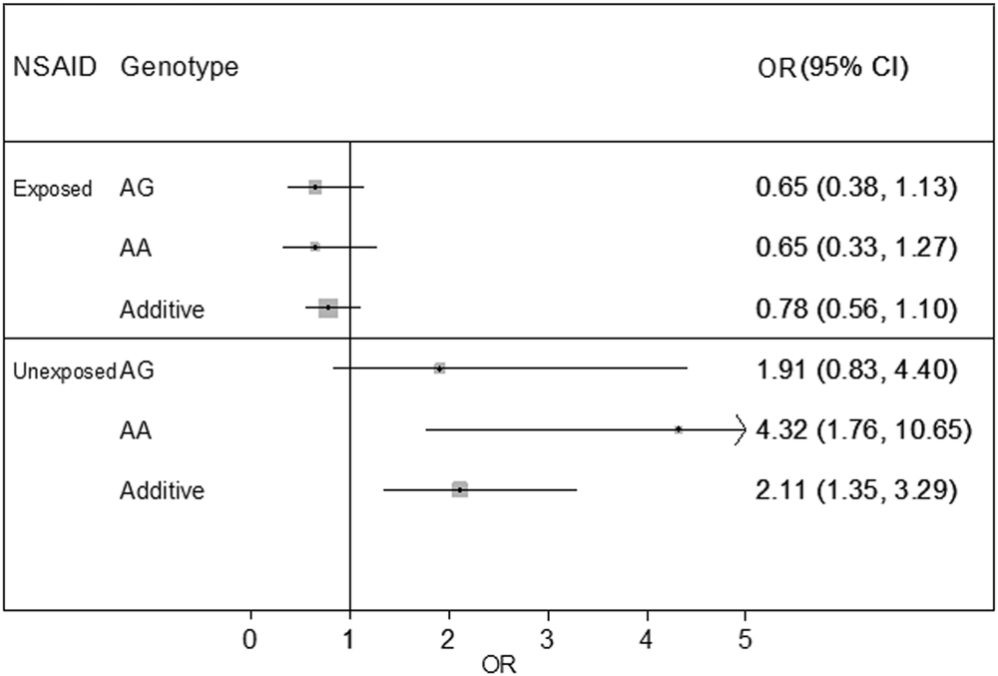
Single locus association analysis of rs5602 stratified by NSAID use. ORs are presented on the X-axis for single SNP association analyses of rs5602 stratified by genotype and NSAID use (yes or no) and modeled with an additive model. The GG genotype is used as the referent genotype for all analyses.

Secondary single SNP association analyses (additive models) stratifying association of the strongest associated SNP (rs5602) by NSAID use (Fig. 2) showed SAB risk was increased with increasing dosage of the A allele for rs5602 (additive model) among women who did not use NSAIDs (OR = 2.11, 95% CI 1.35–3.29) while there was a protective but non-significant effect among those who took NSAIDs (OR = 0.78, 95% CI 0.56–1.10). Genotype-stratified analyses comparing the AA and AG to the GG (referent) genotype showed that women with the AA genotype who did not use NSAIDs were at the highest risk of SAB (AA vs GG unexposed to NSAIDs, OR = 4.32, 95% 1.76–10.65), an effect that was not present among women with the AA genotype who took NSAIDs.

To further evaluate gene variants within *PGIS*, we imputed ungenotyped SNPs within the gene. We observed one SNP that had stronger associations than our strongest associations among genotyped SNPs, rs491025, (OR = 0.28, 95% CI 0.15–0.50, p = 2.74 × 10^−5^). The minor allele ﻿(MA) of rs491025 is the T allele and it had an overall frequency of 0.50, similar to the minor allele frequency (MAF) for rs5602 (MAF = 0.47 overall), suggesting they were in strong LD.

Finally, we evaluated the urinary PGE-M levels for association with the most associated variants identified from interaction analyses using an independent population (Supplemental Table 1). The mean age of the subjects for these analyses was 58.8 years. We observed the highest Beta for the association between rs5602 and urinary PGE-M levels among women (Beta = 0.18, 95% CI 0.01–0.19, p = 0.036). However, a statistically significant association was also observed in males (Beta = 0.10, 95% CI 0.01–0.35, p = 0.038) and overall (Beta = 0.09, 95% CI −0.002–0.19, p = 0.033). These findings have not previously been published.

## Discussion

We have demonstrated an association between a *PGIS* gene variant and risk of SAB. This variant is a potentially functional variant and results in higher urinary PGE-M levels. Among individuals with this variant, NSAID use during pregnancy reduces risk of SAB. This interaction between *PGIS* and NSAID use was statistically significant after a correction for multiple testing. Several nominally significant associations at *PGDR* gene variants were also observed. Our findings suggest that risk for SAB is reduced among women with either one or two copies of the *PGIS* risk A allele who take NSAIDs.

*PGIS* (prostacyclin synthase) is part of the COX-2 signaling pathway (Fig. 1). Studies have shown direct relationships between increased prostacyclin levels and pregnancy complications including SAB^24^. Prostacyclin inhibits platelet activation and is a vasodilator. Inhibition of prostacyclin production causes increased risk for coronary events when taking certain types of NSAIDs^25^. NSAIDs inhibit prostacyclin production in endothelial cells which may result in a prothrombotic state^26^. *PGDR* (prostaglandin D2 receptor) is thought to contribute to the maintenance of pregnancy by controlling Th1/Th2 balance and antigen presentation^27^. It is of note that *PGI2* (Fig. 1, the direct product *PGIS*) and *PGE2* (urinary PGE-M used as a proxy in our study) are antagonistic to each other, where *PGI2* is anti-inflammatory and *PGE2* is proinflammatory^28, 29^. Therefore, our results would suggest that the *PGIS* rs5602 association with SAB risk and higher urinary PGE-M metabolite levels may be due to an imbalance of the *PGI2* and *PGE2* favoring a pro-inflammatory state, which favors a higher risk of SAB^9, 10^. Next steps should include more comprehensive evaluation of all genes and products of the arachidonic acid pathway with attention to use of PG biomarkers to continue to explore functional significance.

The same *PGIS* variant, rs5602, has associated with other diseases. Prior studies have observed associations with *PGIS* rs5602 and rs5629 and risk of myocardial infarction^30, 31^. *PGIS* rs5602 was associated with increased risk for myocardial infarction (G modeled as risk allele) and haplotypes consisting of *PGIS* rs5602-rs5629-rs45498106 were associated with protection from myocardial infarction in Chinese populations^30, 32^. In another study rs5602 associated with protection from myocardial infarction and stroke with an OR = 0.88 (95% CI 0.79–0.96) for myocardial infarction and OR = 0.84 (95% CI 0.73–0.97) for stroke (A modeled as risk allele)^31^. These data suggest the potential for a shared etiology in SAB risk and cardiovascular diseases mediated through pathways involving *PGIS*.

In prior studies evaluating NSAID use in RFTS we observed an interaction by NSAID use and race on SAB risk^6^. NSAID stratified analyses of European Americans (Fig. 2) in the present study showed that subjects with the rs5602 AA genotype were at the highest risk of SAB without taking an NSAID (compared to those with a GG genotype) and experienced significant decreased risk if they took an NSAID. The A rs5602 allele is not common in African ancestry populations with a MAF = 0.02 in the Yoruba population of the HapMap, while it is present with a MAF = 0.50 in the CEU European Ancestry population of the HapMap. We evaluated rs5602 in our African American population from RFTS (data not shown); however, the rs5602 A allele was not present in our SAB cases (MAF = 0) and was present with an MAF = 0.10 in controls. We were therefore not able to replicate the association in our African American population. It is possible that the reason NSAID use interacted with race to modify SAB risk in our prior studies is due to different genetic variants modifying risk in African Americans and European Americans. However, further studies in African ancestry populations are necessary to test that hypothesis.

The RFTS population has strengths that make it well-suited to conduct a study of drug by gene interactions. RFTS obtained detailed and prospective collection of key exposure data regarding medication use during pregnancy, including over-the-counter use, and have DNA for these study participants. We also recruited women either pre-pregnant or in early pregnancy allowing us to capture very early SABs, with a mean gestational age at enrollment of 41 days from LMP. Using Quanto software^33^ we estimated that we had 96% power to detect an ORs of 0.34 under the same parameters and allele frequencies for which we observed our interaction at rs5602 assuming an alpha of 0.05 and 53% for Bonferroni corrected alpha (2.49 × 10^−4^). We acknowledge that we were limited by our sample size and were only able to detect strong effects. Despite this, we observed a result that was statistically significant after a Bonferroni correction for multiple testing. In addition, in our study we were not interested in recurrent pregnancy loss but rather how modifiable exposures (NSAID use) may interact with maternal genetics to modify risk of SAB. Therefore, we cannot conclude whether these variants identified are related to heritable genetic risk factors for recurrent pregnancy loss.

Women and their care providers currently lack evidence with sufficient statistical power and temporal assignment of exposures and outcomes to inform clinical care regarding the consequences of NSAID use during pregnancy. We observed an interaction between NSAIDs and *PGIS* on SAB risk indicating that women who take NSAIDs and who carry *PGIS* rs5602 A allele may be protected from miscarriage. Our findings were further strengthened by the association observed between *PGIS* rs5602 and increased levels of urinary PGE-M that support a potential role for this gene variant and increased level of the proinflammatory cytokine PGE2. Our findings suggest that the inconsistencies in the association between NSAIDs and SAB in prior studies, some studies showing increased risk and other showing no association with NSAID use, may be due to differences in the genetic risk profile of the study populations. Our findings, if independently verified, support that NSAID treatment of pregnant women who carry the A allele for *PGIS* variant rs5602 may reduce risk for SAB. Further investigations of NSAID dose, timing in gestation and validation of our study findings in an independent population is necessary in order to better understand our observed interactions between NSAID use and *PGIS*.

## Methods

### Study Population and Data Collection Protocol

Subjects included in our analyses were obtained from the *Right from the Start* (RFTS) community-based prospective pregnancy cohort. The RFTS study population is not a clinical cohort. We limited our analyses to RFTS participants of European ancestry for whom DNA samples were available. RFTS began enrolling study participants in 2000, and began collecting information about NSAID exposure in 2004. Over time, RFTS has been funded through three major phases with distinctive research questions (RFTS1, 2, 3), and has enrolled participants in Galveston, TX; Memphis, Nashville, Knoxville, and Chattanooga, TN; and the Research Triangle region (Raleigh, Durham, and Chapel Hill) in NC. We limited our SAB cases and comparison group subjects to RFTS 2 and 3, because over-the-counter NSAID use was not ascertained during the interview for RFTS 1. RFTS participants are 18 years or older and did not use assisted reproductive technologies to conceive^34^. Consent was obtained to review all records pertaining to the study pregnancy. Participants were actively recruited and followed from pre-conception or very early pregnancy through the end of pregnancy (mean and standard error of gestational age at enrollment was 41.0 days ± 10.2). Follow-up was conducted to document outcomes. We will make data available upon request.

Participants completed an intake interview at enrollment and a computer assisted telephone interview (CATI) at the end of the first trimester. The CATI was conducted at a mean gestational age of 96 days ± 12.8 days, providing information on history of bleeding or pain, medication use, and exposure to potential confounders in the time since last menstrual period (LMP).

DNA samples were obtained from study participants beginning in 2010 and were collected both prospectively during ongoing recruitment and retrospectively for past participants. All participants with available contact information were approached for collection of DNA samples. Our analyses included all subjects who met our case inclusion and exclusion criteria at the time of genotyping. The institutional review boards (IRB) of Vanderbilt University, Nashville, TN and the University of North Carolina, Chapel Hill, NC approved this study. Informed consent was obtained from all study participants and all methods were performed in accordance with the relevant guidelines and regulations of the study IRB.

#### Pregnancy Outcome Data

Pregnancy outcomes were self-reported and abstraction of medical records was used to verify outcomes. Live births were linked to state vital records to assist in verifying the pregnancy outcomes. Gestational age was estimated from self-reported LMP, which we confirmed was reported with high accuracy in our study population^34^. Women could enroll in RFTS during more than one pregnancy.

SAB cases included in our analyses were subjects with DNA samples and defined as a loss before 20 completed weeks’ gestation. Induced abortions were not included in our SAB case group. For SAB cases we selected the first RFTS pregnancy for which she experienced a loss, for those without losses we use data from their first enrollment. Our SAB cases were limited to subjects with a SAB in their current pregnancy regardless of prior history of loss. We were not interested in identifying heritable factors associated with recurrent pregnancy loss but rather evaluating the role of maternal NSAID use, maternal genetics, and risk of SAB. The comparison group “controls” included both live births and stillbirths, excluding ectopic pregnancies and induced abortions. SAB cases and controls included in our analyses were limited to subjects of European ancestry.

#### Secondary Biomarker Data

Secondary analyses of PGE-M biomarker data used participant data from the *Tennessee Colorectal Polyp Study* (TCPS). The TCPS is a large, colonoscopy-based case-control study of colorectal adenomas. Details regarding data collection, genotyping methods, and data quality control for this study population have been previously described^35, 36^.

### NSAID Assessment and Other Variables

Participants were queried about medications in the intake and first-trimester interviews. NSAID assessment in RFTS has been previously described^1, 6^. We included over-the-counter and prescription medications in statistical analyses. Both interviews included NSAID exposures during the periconceptional period, (e.g. from LMP through six weeks gestation). All NSAID use reported was during the periconceptional period less than six weeks gestation. The primary exposure was classified as any NSAID use versus no NSAID use based on whether the participant reported NSAID use in either interview. For these analyses, women who were classified as non-NSAID users were required to answer three or more of the drug questions regarding NSAID use and report no NSAIDs were used. Aspirin-only users were not included as NSAID users.

Maternal characteristics and obstetric history were also recorded. These included: height, weight, maternal age, race/ethnicity, diabetes status, parity, gravidity, induced abortion history, study site, and smoking status (current or not current smokers). Information on these characteristics was obtained from either the study ultrasound visit or first-trimester interview.

### DNA Extraction and Genotyping

DNA for RFTS saliva samples was extracted using Oragene DNA (Genotek Inc., Ontario, Canada) manufacturer recommended DNA extraction procedures. We genotyped 384 SNPs (100 ancestry informative markers [AIMs]and 284 haplotype tag [htSNPs] and candidate SNPs) using the Illumina BeadXpress® System (Illumina, Inc., San Diego, CA) custom array.

This experiment was part of a larger genotyping experiment that also included a small subset of African Americans from RFTS. AIMs were selected from three populations from the HapMap phase III database (www.hapmap.org) (Yoruba (YRI), Centre d’Etude du Polymorphisme Humain (CEU) European ancestry, and Hispanic (MEX)) to estimate the proportion of ancestry arising from African, Hispanic, and European populations in our study participants^37^. SNPs were selected to have maximal allele frequency differences between populations and be 1–5 mega base pairs from selected candidate genes in order avoid associations of outcomes with AIMs. The 284 SNPs haplotype tag and candidate SNPs came from 27 NSAID drug metabolism genes. The genes included: CYP450 genes (CYP1A1, *CYP1A2, CYP2A6, CYP2B6, CYP219, CYP2C8, CYP2C9, CYP2D6, CYP2E1, CYP2C19, CYP3A4, CYP3A5, CYP2A7P*), genes specific to cyclooxygenase (*COX-1 (PGS1)*, *COX-2 (PGS2), PGER1, PGER2, PGER3, PGER4, PGIR, PGDS, TBXA2R, PGIS* (*CYP8A1*), *PGIR*, *PGDR*), lipoxygenase pathways (*ALOX15*)), and other downstream factors (*IL-6*). SNPs selected from candidate genes included a combination of htSNPs selected from the HapMap (www.hapmap.org) using CEU samples to select htSNPs for European Americans and putative functional candidate SNPs selected from the literature. Candidate SNPs were forced into the Tagster htSNP selection algorithm available at SNPinfo (www.snpinfo.niehs.nih.gov/snptag.htm), an iterative approach for choosing a minimum set of htSNPs for study subjects with admixed ancestry^37, 38^. Using this approach, consensus htSNPs were chosen after forcing in candidate SNPs to provide coverage (r^2^ greater than or equal to 0.8) of common variation (minor allele frequency [MAF] greater than or equal to 0.10) for all candidate genes extending ± 5 kb from the Reference Sequence database gene boundaries.

All samples and SNPs had genotyping call rates of ≥95%. We limited analyses to SNPs to those with MAF ≥0.05 resulting in 201 SNPs included in analyses after quality control (QC).

### Statistical Analysis of Genotyping Data

For analysis of genotype data, tests for deviations from Hardy Weinberg Equilibrium (HWE) were performed using PLINK statistical software^39^. Statistical significance for these analyses was determined using p values from Fisher’s exact tests. Descriptive statistics of demographic data were expressed as means and standard deviations for continuous covariates and as frequencies and proportions for categorical data, and compared between cases and controls (women who had an SAB versus live birth) with unadjusted linear or logistic regression using STATA 11.0 statistical software (College Station, TX).

Pairwise linkage disequilibrium (LD) was characterized using the summary statistics D’ and r^2^, and haplotype frequencies were calculated using HaploView software^40^. Haplotype blocks were assigned, using the D’ confidence interval algorithm created by Gabriel *et al*.^41^. STRUCTURE software v2.3.3 was used to evaluate population stratification and estimate proportions of ancestry using AIMs^42^. STRUCTURE applies a Bayesian non-hierarchical clustering method and was examined under different assumptions of the number of ancestral clusters ranging from K = 1–3 with and without any pre-assignment of ancestral affiliation.

Logistic regression was used to evaluate the interaction between NSAID use (yes/no) in the first trimester and SNPs on SAB risk (SAB versus live birth) using PLINK statistical software^39^. Analyses were performed using an additive genotypic model with minor allele coded as the risk allele. Analyses were conducted adjusting for NSAID use, maternal age, BMI, SAB history, and ancestry proportion. A Bonferroni correction for multiple testing was used to determine the threshold for statistical significance. We also performed secondary analyses limiting SAB cases to those with early losses (<10 weeks gestation) and late losses (≥10 weeks gestation) to assess the potential impact on NSAID use on gestational age at loss. We also preformed secondary analyses excluding the salicylate (NSAIDs with aspirin as a secondary active ingredient) NSAID class.

We also imputed genotypes at additional variant sites in the regions of interest by prephasing haplotypes with SHAPEIT^43^ and using IMPUTEv2^44^ software to impute ungenotyped SNPs. We used the entire panel from the 1000 genomes reference population (build 37, 2013). Interaction analyses of NSAID and imputed SNPs were analyzed using ProbABEL software and modeled adjusting for NSAID use, maternal age, BMI, SAB history, and ancestry^45^. Data used in analyses described are available upon request.

### PGE Metabolite Assays

To quantify endogenous PGE_2_ production, urinary PGE-M (11 alpha-hydroxy-9,15-dioxo-2,3,4,5-tetranorprostane-1,20-dioic acid) level was measured using liquid chromatography/ tandem mass spectrometric method previously described by Murphey *et al*.^46^. The measurement of urinary PGE-M, developed at Vanderbilt University, provides the most accurate approach to assess the endogenous production of PGE_2_ in humans. Briefly, 0.75 mL of urine per patient was titrated to a pH of 3 using 1 mol/L HCL and then 0.5 mL of methyloxime HCl. Methoximated PGE metabolites were extracted and applied to a C-18 Sep-Pak (Waters Associates, Milford, MA) and eluted with 5 mL ethyl acetate. An internal standard of [^2^H_6_] *O*-methyloxime PGE-M was added. Liquid chromatography was performed on a Zobrax Eclipse XDB-C18 column attached to a ThermoFinnigan Surveyor MS Pump (Thermo Finnigan, San Jose, CA). For endogenous PGE-M, the predominant product ion *m*/*z*336 representing [M-(OCH_3_ + H_2_O)]^−^ and the analogous ion *m*/*z*339 representing (M-OC[^2^H_3_ + H_2_O]), for the deuterated internal standard, were monitored in the selected reaction monitoring (SRM) mode. Quantification of endogenous PGE-M utilized the ratio of the mass chromatogram peak areas of the *m*/*z*336 and *m*/*z*339 ions.

### Statistical Analysis of PGE Metabolite Assays

Association analyses of PGE metabolite levels were conducted using log-transformed PGE-M using previously genotyped and imputed SNPs. These analyses were limited to the top associated SNPs (statistically significant after multiple testing correction) identified from interaction analyses using RFTS samples. The QC and imputation of the genetic data have been previously described^36^. Linear regression was conducted using SNPtest^47^ software adjusting for age and sex. Recent NSAID use was missing too often to include as a covariate in models. Secondary analyses were also performed stratifying by sex and adjusting only for age.

## Electronic supplementary material

Supplementary Information
