# Supplementary material for: Nonsteroidal Anti-inflammatory Drug Interaction with Prostacyclin Synthase Protects from Miscarriage
Source: Sci Rep. 2017 Aug 29;7:9874. doi: 10.1038/s41598-017-10150-2 (PMC5575303; doi:10.1038/s41598-017-10150-2)
Supplement: Supplementary file 1 — Supplementary Information [file 41598_2017_10150_MOESM1_ESM.pdf]

Supplemental Information

Nonsteroidal Anti-inflammatory Drug Interaction with Prostacyclin Synthase Protects from Miscarriage

Digna R. Velez Edwards,[1,2,3,4*] Todd L. Edwards,[2,4,5,7] Michael J. Bray,[4] Eric Torstenson,[4] Sarah Jones,[2] Martha J. Shrubsole,[1,2,5,7] Harvey J. Muff,[5,6,7] and Katherine E. Hartmann,[1,2,3]

[1]Vanderbilt Epidemiology Center, [2]Institute for Medicine and Public Health, [3]Department of Obstetrics and Gynecology, [4]Vanderbilt Genetics Institute, [5]Division of Epidemiology, [6]Division of General Internal Medicine and Public Health, Vanderbilt University, Nashville, Tennessee, 37203, [7]GRECC, Department of Veterans Affairs, Tennessee Valley Healthcare System, Nashville, Tennessee

*Corresponding author: digna.r.velez.edwards@vanderbilt.edu

A.

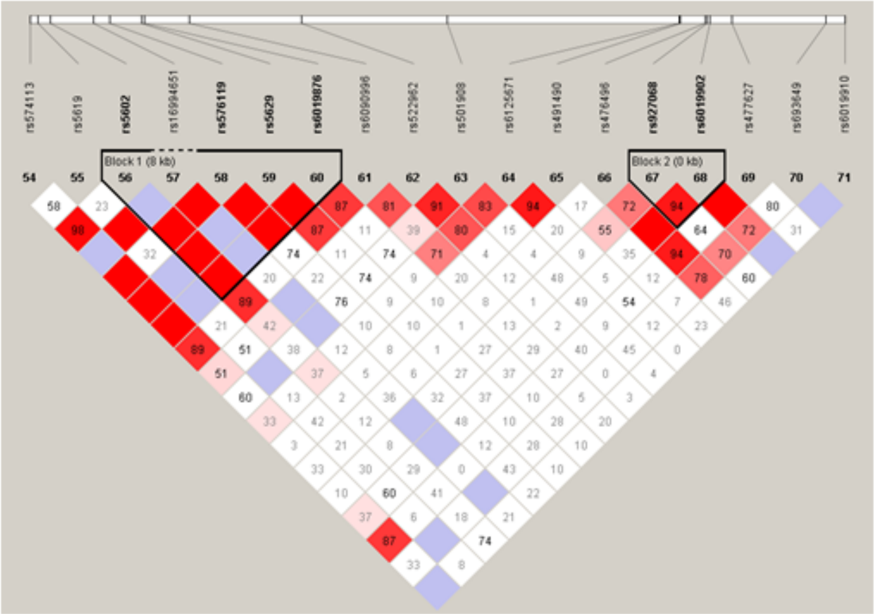
rs574113
rs5619
rs5602
rs16994651
rs576119
rs5629
rs6019876
rs6090996
rs522962
rs501908
rs6125671
rs491490
rs476496
rs927068
rs6019902
rs477627
rs693649
rs6019910
Block 1 (8 kb)
Block 2 (0 kb)
54 55 56 57 58 59 60 61 62 63 64 65 66 67 68 69 70 71

B.

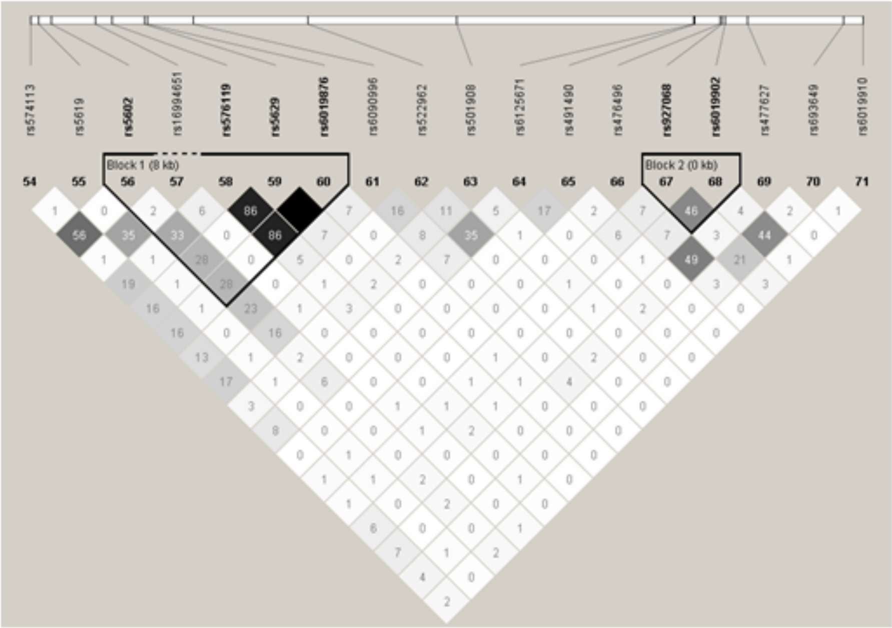
rs574113
rs5619
rs5602
rs16994651
rs576119
rs5629
rs6019876
rs6090996
rs522962
rs501908
rs6125671
rs491490
rs476496
rs927068
rs6019902
rs477627
rs693649
rs6019910
Block 1 (8 kb)
Block 2 (0 kb)
54 55 56 57 58 59 60 61 62 63 64 65 66 67 68 69 70 71

C.

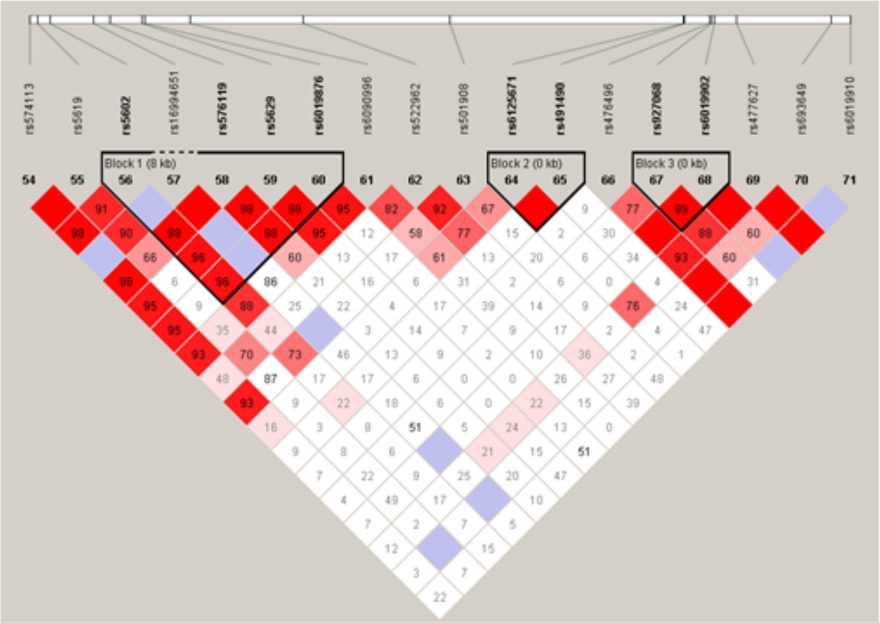

D.

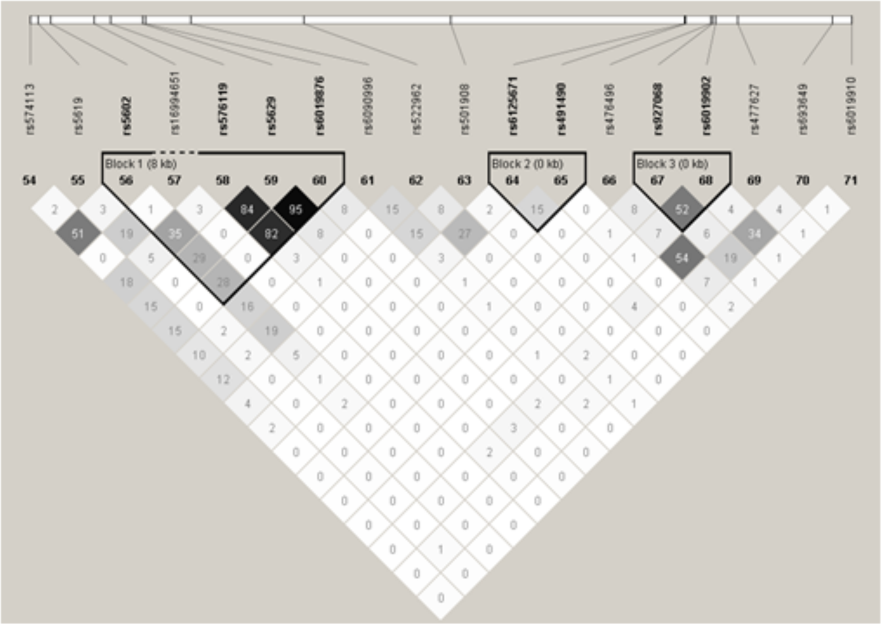

Supplemental Figure 1. Linkage disequilibrium in *PGIS* in cases and comparison group. Linkage disequilibrium (LD) plots are presented for SAB cases and comparison group including both D' and r^2. All figures are oriented 5' to 3', right to left, relative to the gene orientation on the

minus strand. D’ (shades of red) and r^2 (shades of black) are indicated in percentages within squares in the LD plots, with solid blocks without numbers indicating D’ and $r^2 = 1$. Strong LD is indicated by red or dark gray, while pink and light gray and white indicate uninformative and low confidence values, respectively. LD Blocks were created in HaploView that creates 95% confidence bounds on D’ considered being in strong LD where 95% of the comparisons made are informative.

Supplemental Table 1. PGE-M single SNP association analyses of strongest association SNPs from Table 2

Gene	rsID	Model	N	RA	RAF	BETA	95% CI		P
							Lower	Upper	
PGIS	rs5602	**Overall**[1]	610	A	0.48	0.09	-0.002	0.19	0.033
		**Males**[2]	453	A	0.46	0.10	0.01	0.35	0.038
		**Females**[2]	157	A	0.51	0.18	0.01	0.19	0.036

Chr-chromosome; BP-base pair; RA-risk allele; RAF-risk allele frequency; CI-95% Confidence Interval.
[1]Models adjusted for age and sex
[1]Models adjusted for age
